# World’s First Long-Term Colorectal Cancer Model by 3D Bioprinting as a Mechanism for Screening Oncolytic Viruses

**DOI:** 10.3390/cancers15194724

**Published:** 2023-09-26

**Authors:** Colin McGuckin, Nico Forraz, Clément Milet, Mathieu Lacroix, Yordan Sbirkov, Victoria Sarafian, Caroline Ebel, Anita Spindler, Véronique Koerper, Jean-Marc Balloul, Eric Quéméneur, Cécile Zaupa

**Affiliations:** 1 CTIPharma Department, Cell Therapy Research Institute, CTIBIOTECH, Bat A16, 5 Avenue Lionel Terray, Meyzieu, 69330 Lyon, France; nico.forraz@ctibiotech.com (N.F.); clement.milet@ctibiotech.com (C.M.); mathieu.lacroix@ctibiotech.com (M.L.); 2 Department of Medical Biology and Research Institute, Medical University of Plovdiv, 4002 Plovdiv, Bulgaria; yordan.sbirkov@mu-plovdiv.bg (Y.S.); sarafian@abv.bg (V.S.); 3 Transgene, Illkirch-Graffenstaden, 67400 Strasbourg, France; ebel@transgene.fr (C.E.); spindler@transgene.fr (A.S.); koerper@transgene.fr (V.K.); balloul@transgene.fr (J.-M.B.); quemeneur@transgene.fr (E.Q.); zaupa@transgene.fr (C.Z.)

**Keywords:** 3D bioprinting, colorectal, oncolytic virus, longitudinal

## Abstract

**Simple Summary:**

Although many new cancer drugs look like they are working in the laboratory, they fail when tested on real people. This is often because the cancer cell models they are tested on are short-term, but cancer grows in people over a longer period, and things change over time. This is not a criticism of cancer laboratories, but more a realism of the limits of scientific research. This is why we developed this longer-lasting model of colorectal cancer that has moved around the body. At the same time, we used this model to test next-generation delivery of chemotherapy drugs to tumors with an advanced targeting virus. All levels of cancer research have value, both short-term and long-term, but the longer strategies need to catch up, so this study demonstrates what can be achieved with 3D bioprinting tumor models and testing viruses transformed for good.

**Abstract:**

Long-term modelization of cancer as it changes in the human body is a difficult goal, particularly when designing and testing new therapeutic strategies. This becomes even more difficult with metastasis modeling to show chemotherapeutic molecule delivery directly to tumoral cells. Advanced therapeutics, including oncolytic viruses, antibody-based and cell-based therapies are increasing. The question is, are screening tests also evolving? Next-generation therapeutics need equally advanced screening tests, which whilst difficult to achieve, are the goal of our work here, creating models of micro- and macrotumors using 3D bioprinting. We developed advanced colorectal cancer tumor processing techniques to provide options for cellular expansion, microtumor printing, and long-term models, which allow for the evaluation of the kinetics of penetration testing, therapeutic success, targeted therapies, and personalized medicine. We describe how we tested tumors from a primary colorectal patient and, applying 3D bioprinting, matured long-term models for oncolytic metastatic screening. Three-dimensional microtumors were kept alive for the longest time ever recorded in vitro, allowing longitudinal studies, screening of oncolytic viruses and realistic modelization of colorectal cancer. These 3D bioprinted models were maintained for around 6 months and were able to demonstrate the effective delivery of a product to the tumoral environment and represent a step forward in therapeutic screening.

## 1. Introduction

Applications of new technologies in cancer research have advanced rapidly with molecular technologies, but cellular models are often limited by the length of time the tumoral cells survive in vitro and the research endpoint in the experiment. Many models are sacrificed in the shorter term in hours or days, and some even reach a few weeks, but long-term models allowing continued analysis and readouts are much less frequent. There has been increased discussion of bioconvergence and additive manufacturing, where several technologies are integrated together to achieve a longer-term goal. In medical science, the separation of technologies and research has often hindered real progress, not least due to the costs involved, which suggests that a true additive manufacturing advantage would help. This strategy becomes increasingly important in cancer research with the development of advanced viral, antibody or cellular treatment strategies.

**Figure 1:**
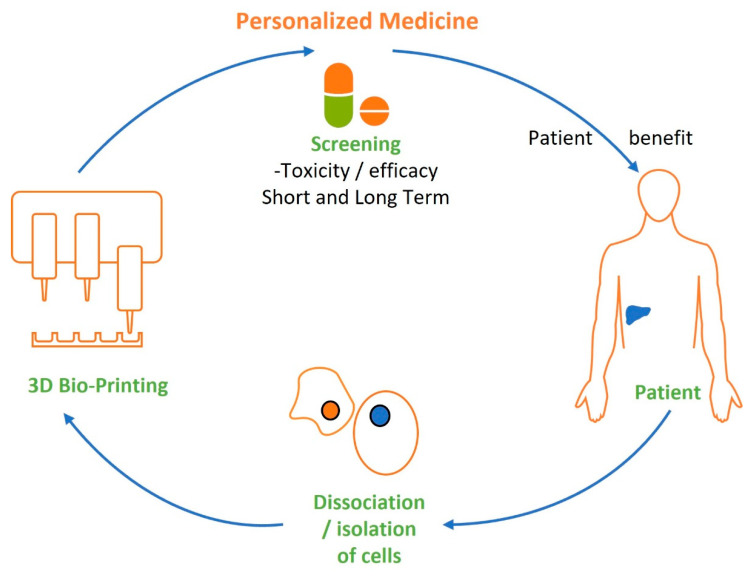
Personalized medicine in principle: 3D bioprinting is now a definable part of the personalized medicine process with a clear role for creating tissues for drug screening and development, and movement towards therapeutic benefit.

Personalized medicine is another term often raised and is an important concept (Figure 1). However, the reality has been rather slow to develop. Global medical systems tend to offer solutions to groups of people rather than individuals, as cost-effective solutions. This grew strongly after the Second World War, when centralized hospitals and medical services rapidly became the norm. However, whilst this allowed population growth and an increase in the average human lifespan, it could not have predicted the needs of a global population of 8 billion increasingly older people. Increases in lifespan come with the reality that the human body is not a perfect organism, with mutations and degradation of systems accumulating over time.

So, what is the solution? Indeed, the combination of technologies and personalized medicine can help to create new treatments not thought of before, and advanced screening systems are a significant part of this journey [1].

Three-dimensional bioprinting is an additive manufacturing strategy for placing cells and/or molecules into a bioink support that is spatially printed in the x, y and z axes. Previously, this was employed in 3D rotating bioreactors by self-assembly with varying results [2]. In 3D printing, however, if the bioink is sufficiently constructed to stay in one place, either by its own structural properties or after crosslinking with light or chemical-based stimulation, then the cellular products can be located in niches inside the construct designed with readily available software. Many bioprinters exist, most based on the original patents for 3D printing of plastics, which have now expired. Some are based on pneumatic, mechanical, or heat-based extrusion principles, but essentially, they are designed to deposit live tissue or cellular material in the 3D axes [3].

However, 3D bioprinting only works if researchers can not only process cells and tissues sufficiently but also keep them alive long enough with the correct growth media. Having spent many years developing procedures to harvest and process cancer tissues, we understand that this stage and indeed the biobanking of those products are significant processes.

In the work presented here, we show how this works in reality, from the harvesting of tissues and preparation of cells to the 3D printed cancer screening product, which is used to monitor the growth and development of tumors in the laboratory. For this paper, we have focused on colorectal cancer (CRC), which remains one of the most serious malignancies worldwide [4,5,6]. Rates of metastasis in this cancer are serious, and the speed of diagnostics before metastatic events is poor [7,8].

CRC is the third most common type of cancer and the third leading cause of death among all malignancies, with over 50,000 mortalities per year only in the U.S. [5]. The number of newly diagnosed patients increases every year, and it is somewhat of concern that this particular cancer is becoming more and more common among young adults [6]. Metastatic disease is the most common cause of death in CRC. The primary site of tumor spread is usually the liver, and it is estimated that about half of all CRC patients will develop such metastases. Remission rates in these cases, due mainly to the altered biology of these metastatic cells compared to the primary tumor, are unfortunately only around 15–20%, and the heterogeneity of the clonality and indeed the potential mutagenesis during growth are part of the problem [7,8,9]. Therefore, creating a highly reproducible and physiologically relevant platform that would allow the study of the dynamics of these aggressive and difficult-to-treat cells, and further, the testing of novel therapeutics, has been urgently required for some time.

The aim of this paper was to create a model that was simple enough to be cost-effective but complex enough to be able to survive long-term as we move to have screening systems that can mimic what happens in the patient. The model also needed to be responsive and physiologically react to treatment in a measurable capacity. Many 3D models, mostly static or self-assembling, have been applied in cancer research with limited data endpoints, including spheroids and organoids [10,11,12]. The creation of real 3D models is important to advance beyond these simple organoid models which are variable at best and over-labeled as 3D models in most cases [13,14]. Although many and varied models are required in cancer research and each has its place, the current limitations of organoids are that the assembly of cells is limited in cell numbers and often is a passive aggregation that has no realistic physiology. Organoid models tend to have limitations on time of growth or short endpoints and therefore limited output of testing and information available [15,16,17,18].

The advantage of creating long-term 3D bioprinted models is that this would allow for side-by-side patient monitoring to assess if a tumor is mutating, since mutagenesis must be considered, and potentially to pre-test chemotherapeutic combinations and doses appropriate for the treatment of the patient. Whilst that may be difficult for some cancers, it is a realistic and important opportunity for colorectal cancer treatment [19,20].

Further, a significant advantage of long-term truly 3D tumor models is penetration testing, which remains one of the biggest failure areas for new chemotherapeutic strategies. The inability of some therapeutics to gain entry into the tumor microenvironment due to the self-encapsulation of the tumor by fibrotic digestion, immunological circulation kinetics or dysregulation of the surrounding “healthy” tissues remains a barrier to successful treatment. Therefore, 3D models can help to evaluate many aspects of drug kinetics and patient specificity that other systems fail to do [21,22].

The cost of developing cancer strategies is severe, not least at the animal model level, where many treatments fail to progress. The creation of in vitro humanized 3D models could create a significant advantage over and perhaps in the future replace animal models in drug and toxicology testing, creating more specific solutions in cancer whilst at the same time reducing overall costs, which ultimately could lead to lower patient treatment costs and a wider spread of global cancer therapy support.

## 2. Materials and Methods

### 2.1. Sample Collection and Processing

The official biobank of the IMODI cancer consortium (https://imodi-cancer.org/) is located on-site and is authorized by the French government to collect human cancer samples with authorization under local, national, and European ethical guidelines and regulations. The sample provided was delivered at 4 °C to the biobank within 48 h of collection from the donor. Available donor details allowed under French anonymization laws are listed in Table 1.

**Figure 2:**
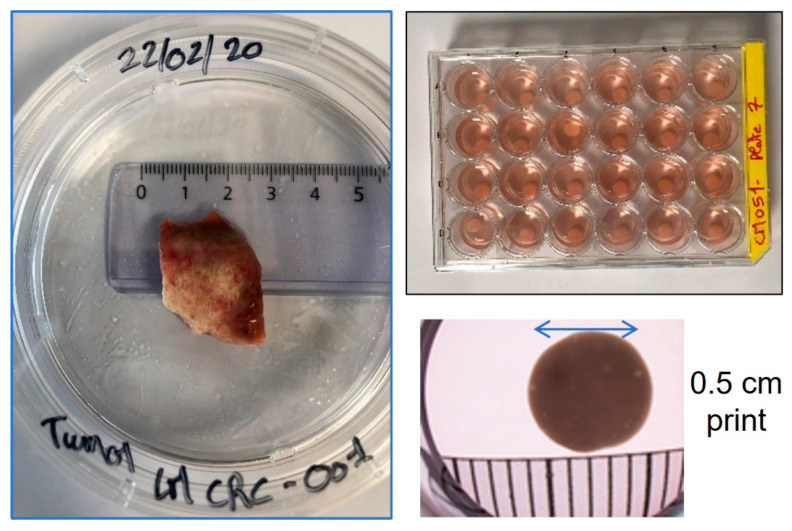
Patient tumor characteristics and resulting printed tumor models. Clockwise: Original tumor donation piece. Printed identical microtumor plate after 3D bioprinting in 24 well format. Full microtumor model with designed size of 0.5 cm diameter and 0.05 cm thickness.

Sample sizes available following surgery are categorized for diagnostics before research as expected, so the final tumor availability is often a limitation of the work. For this reason, every part of the tumor is kept for use either in direct research or later genetic-related projects. A physical representation of the tumor and the types of 3D printed models available after processing are given in Figure 2.

**Figure 3:**
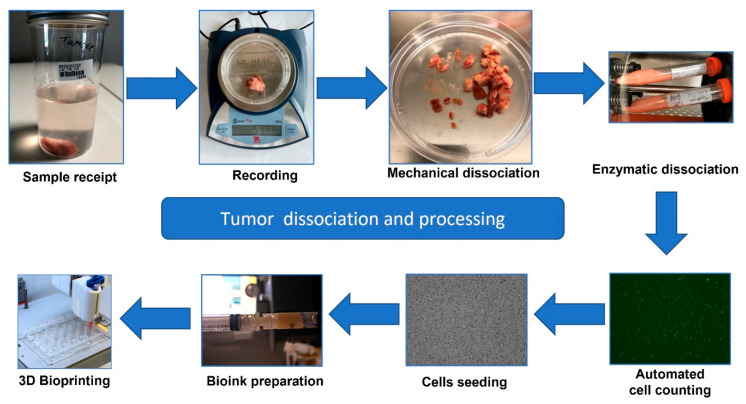
Visual representation of the laboratory flow for tumor dissociation and processing for 3D bioprinting. The process of tumor receipt, logging and dissociation, using manual slicing, followed by enzymatic dissociation on a tissue roller is represented. Resulting free cells can either be printed immediately or recultured for selection of tumoral cells, cancer-associated fibroblasts and, if metastatic, host organ tissue.

Colorectal liver metastasis cells were isolated from the primary human sample from the patient as follows: First, the tumor was manually dissociated into small pieces of approximately 1 mm^3^ before enzymatic dissociation using a 1:5 solution of Collagen IV and Dispase (StemCell Technologies, Saint-Egrève, France). After incubation at 37 °C for 1.5 h, cells were filtered through a 70 µm filter (Miltenyi, Bergisch Gladbach, Germany) and centrifuged at 250 G for 7 min (ThermoFisher Heraus X3R, St Quentin-Fallavier, France). The count and viability of all cells were tested using a LUNA-FL dual fluorescence cell counter (Logos Biosystems, Villeneuve d’Ascq, France) (Figure 3).

### 2.2. Cell Culture and 3D Bioprinting

**Figure 4:**
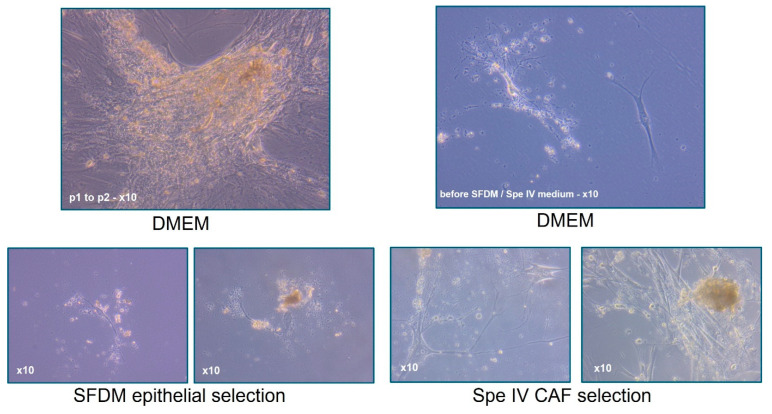
Tumor amplification and selection of epithelial-like cells and cancer-associated fibroblasts. Selection and amplification of cells are nutrient-media-dependent. Although all cells can be amplified at different speeds, selection in cell-specific media promotes particular cell types, allowing choices in cellular expansion and later model creation. In addition, the cells are then also stored in our biobank for later genetic studies.

The expansion of disaggregated cells was performed in a coculture media of DMEM/F12 Glutamax (Gibco, Thermofisher, St Quentin-Fallavier, France) with 10% FBS (Hyclone, Thermofisher) to maintain both populations of primary cancer cells and microenvironmental cancer-associated fibroblasts. However, it was also possible to show a selective expansion of either epithelial-like cancer cells or cancer-associated fibroblasts by growth in Serum Free Defined Media (SFDM) or SPE IV media, respectively (both adapted media produced at CTIBIOTECH, Lyon, France). (Figure 4).

Once expanded, cells were removed from the culture with gentle dissociation using Enzyme Express (1X) TrypLE™ (Gibco, Thermofisher).

Three-dimensional bioprinted microtumor models were designed and “software-sliced” beforehand on a computer using SketchUp (https://www.sketchup.com/, Westminster, CO, USA) paid and Slic3r (https://slic3r.org/, Rome, Italy) opensource software, respectively, in order to generate a G-code file to transfer to the bioprinter. Amplified cells and corresponding culture media were separately mixed with a bioink suitable for tumor development at a concentration of 15 million/mL and 3D printed (Bio X, CELLINK, Goteborg, Sweden), with RGD bioink (catalog # IK1020100301, composed of alginate with covalently bound RGD and nanofibrillar cellulose, with a viscosity of 3–20,000 Pa/s and shear rate 0.002–500 1/s (from the manufacturer’s specification sheet)). Bioinks and the bioprinting laboratory were gas-controlled and temperature-controlled before mixing with cellular material and nutrient media. Printing was performed in 12- or 24-well culture plates according to our optimization protocols, using sterile 3 mL cartridges (catalog # CSC010311101, CELLINK, Sweden). The cell–bioink mixture was then extruded through a 25 gauge (0.25 mm) high-precision nozzle (catalog # NZ3220005001, CELLINK, Sweden) at 8–10 kPa pressure, 15 ms speed (with 20 ms preflow delay), in standard 24-well cell culture plates. Multiple cartridges were used as appropriate until sufficient models were created for experimentation. Bioprinted models were then crosslinked for 1–5 min with CaCl_2_ (catalog # 1010006001, CELLINK, Sweden), washed with PBS and left with 2 mL DMEM/F12 medium 10% SVF in the incubator. Media were replaced every few days throughout experimentation unless the protocol for further treatment required a difference. Bioprinted models were kept growing for between 2 days and more than 5 months.

### 2.3. Cell Viability and Microtumor Analysis

Testing of the model was carried out between Day 2 and Day 169 directly by transmitted light and fluorescence labeling.

The viability of the model was assessed with standard live–dead analysis: Briefly, cell viability within the 3D models was assessed using the LIVE/DEAD™ kit (Invitrogen™, Waltham, MA, USA). This kit stains live cells with Calcein AM and dead cells with ethidium homodimer 1. The staining can be detected using an inverted fluorescence microscope (Nikon Eclipse Ti-S, Tokyo, Japan), with live cells expressing green fluorescence (excitation: 494 nm, emission: 517 nm) and dead cells expressing red fluorescence (excitation: 528 nm, emission: 617 nm). Macroscopic follow-up of microtumor growth and viability was performed using a stereo fluorescence microscope (Nikon SMZ18, Tokyo, Japan) with transmitted light and green fluorescence (excitation: 494 nm, emission: 517 nm) following staining of viable cells with Calcein AM (Invitrogen™, Waltham, MA, USA).

Histological assessment was performed using paraffin-embedded 3D bioprints after fixation in 4% formaldehyde−16.7 mM CaCl_2_ solution. Paraffin-embedded 3D bioprints were then cut in 4 µM sections and mounted on slides. Sections were deparaffinized and hydrated, and then hematoxylin and eosin (H&E) and Masson–Goldner trichrome (Bio-optical) staining were carried out for pathomorphological assessment. Immunohistological analyses were carried out using a Bond RXM (Leica, Wetzlar, Germany). After deparaffination and rehydration, epitope retrieval was performed using Bond epitope retrieval solution 2 (AR9640, Leica). Endogenous peroxidase activities were blocked by incubating sections in hydrogen peroxide block solution (TA-125-H2O2Q, LABVISION, Thermofisher, St Quentin-Fallavier, France) for 10 min at room temperature. Sections were saturated with 10% goat serum for 20 min. Primary rabbit antibody anti-Ki67 (0.2 µg/mL-LS-B13463 LS-Bio) was incubated for 1 h at room temperature and then revealed using Novolink anti-Rabbit polymer (RE7161, Leica) followed by a Tyramide System Amplification step (TSA-Fluorescein, SAT701001EA, Akoya, Marlborough, MA, USA) and a counter-staining step using bis-Benzimide Hoechst3328 (B-2883, Sigma-Aldrich, St Quentin-Fallavier, France).

### 2.4. Hypoxia Assessment of 3D Bioprinted Microtumors

Hypoxic areas of microtumors were investigated using labeling with pimonidazole hydrochloride (PIMO), which forms stable covalent adducts with thiols in hypoxia. The staining was performed using Hydroxyprope Omni Kit (Hydroxyprobe, Burlington, VT, USA) according to the manufacturer’s protocol. Briefly, models were incubated for 3 h with growth medium supplemented with 200 µM of PIMO; then, after two PBS washes, they were fixed overnight with 4% formaldehyde and CaCL_2_ (16.7 mM). Next, models were dehydrated, paraffin-embedded and cut into 4 µm sections. The slides were treated as described previously with the Bond RXM except for the epitope retrieval, which was performed with Bond epitope retrieval solution 1, and the primary antibody, which was rabbit IgG anti-Pimonidazole (1/1000; PAb2627, Hydroxyprobe).

### 2.5. Oncolytic Viral Infection and Analysis of Microtumors

Microtumors were infected with an mCherry-expressing oncolytic virus, which is derived from vaccinia virus [23]. The virus carries deficient thymidine kinase and ribonucleotide reductase genes which renders it dependent on cellular enzymes for its DNA replication, and thus, the oncolytic virus replicates preferentially in tumor cells, which frequently overexpress enzymes involved in nucleotide synthesis. The virus (5.104 or 5.105 pfu (plaque forming units)) was added to microtumors in the culture medium (DMEM/F12 10%SVF) in a 24-well plate. The culture medium was changed every 2 or 3 days. Infection of microtumors was monitored for 21 days by following mCherry expression from the oncolytic vaccinia virus (oVV), i.e., observing red fluorescence (Ex 500/24 Em 542/27) using a stereo fluorescence microscope (Nikon SMZ18, Tokyo, Japan). At 25 days after infection, microtumors were fixed overnight with 4% formaldehyde and CaCl_2_ (16.7 mM) and treated as described previously to perform Masson–Goldner trichrome staining.

## 3. Results

### 3.1. Patient-Derived Cellular Expansion from Small Donations Can Be Selective or Multi-Phenotype

Following surgical tumor removal (Figure 2), there are many requests for pieces of the tissue from diagnostics for research. Every part of the tumor is a precious resource. For this reason, we designed gentle tumor processing starting with mechanical followed by enzymatic disaggregation. This was carried out as soon as possible after surgery (Figure 3). Although immediately printing or analyzing the resulting cells is possible and has been done in the past, we opted to carry out expansion to investigate the efficacy of creating more tissues to allow more experimentation and make every piece of the donated tumor useful (Figure 4). Initial expansion in the DMEM media was useful for the early stages of allowing the cells to relax after disaggregation and remove any unwanted debris but was not sufficient for the expansion of the cells. For this reason, we employed a more specific expansion of epithelial-like cells in SFDM media and cancer-associated fibroblasts (CAFs) in SPE IV media (Figure 4).

At different cellular passages, samples were placed into our biobank for later referencing and/or expansion. Following early passages, sufficient cells were expanded in order to carry out 3D bioprinting. In around 1 h, it was possible to make 173 initial and identical models with the first printing (Figure 2).

### 3.2. Development of Microtumors for Short-Term and Longitudinal Studies Is Achieved with 3D Bioprinting

**Figure 5:**
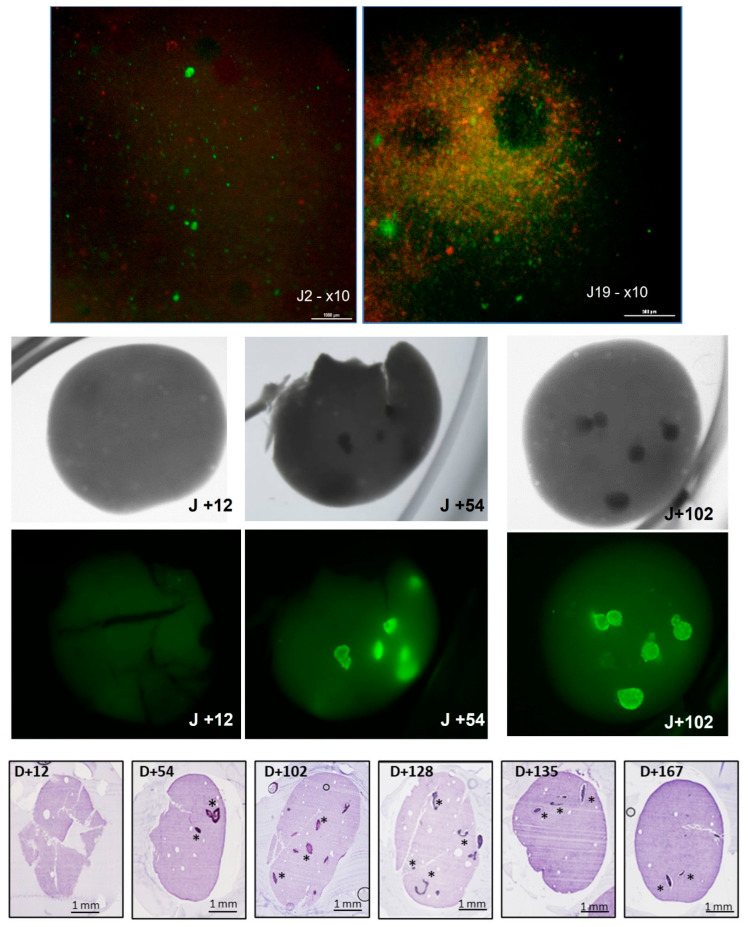
Microtumor development can be achieved over time and allows for research at different stages of tumor development. Upper panels show the development of single cells into groups of microtumoral composites witnessed over 19 days on standard microscope analysis by live–dead analysis. Middle panels show the transmitted light and calcein-labeled composites of microtumors forming stable groups. Lower panels show, up to 167 days, stable microtumors formed in the 3D bioprinted models through HES histology and thin-slice imaging. *, example locations of tumors.

Part of the importance of creating these models was to have options, from short- to medium- to long-term. Although the speed of cellular growth in 2D can give some information, it fails to give an understanding of how cancer cells react in a 3D environment. Over the initial days, the speed of growth of the models was observed with standard live–dead testing (Figure 5 upper panels). Interestingly the speed of growth was higher in the 3D environment than in the 2D expansion culture and by the second week, it was possible to see not just individual cells within the bioprint, but actual structures forming.

By using transmitted light and staining of the cells, it was possible to non-invasively track the development of tumor growth with time (Figure 5 central panels).

With this and histological analysis (Figure 5 lower panels, H&E staining), it was possible to view the development of tumors over the longitudinal axis. Amazingly, the viability and survival of the tumors continued not for weeks, but for months. This was an important hurdle to overcome in developing a personalized medicine model since it allows the comparison of tumor growth in patients and potentially in the future for genetic screening for further mutations.

### 3.3. Replication of In Vivo Heterogeneous Tumor Areas with 3D Bioprinted Microtumors

**Figure 6:**
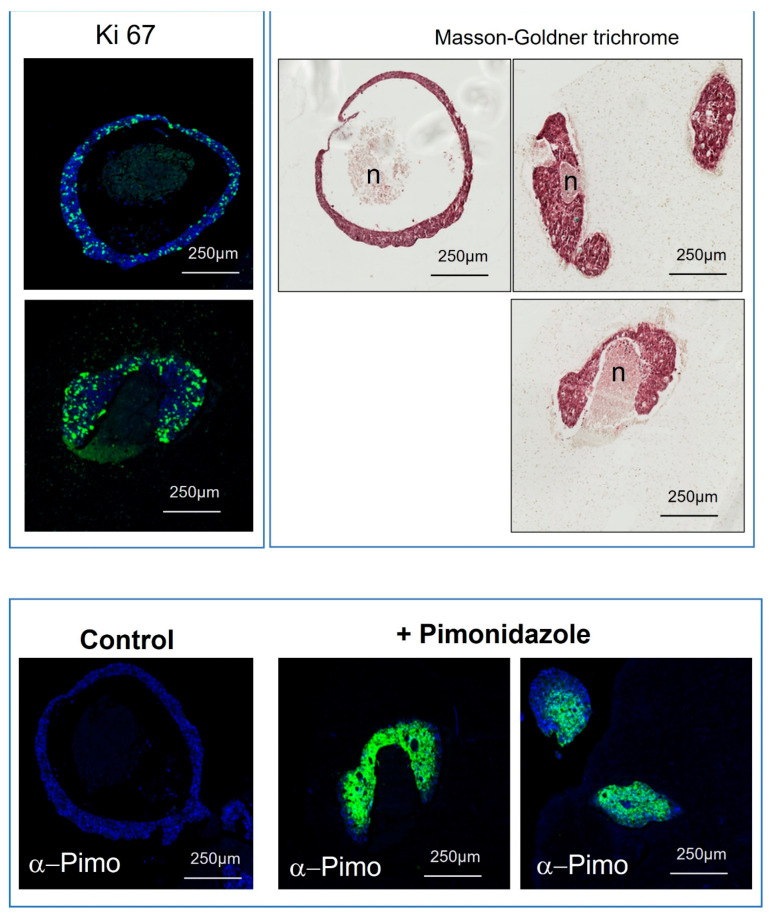
Microtumor characterization 4 months after bioprinting. Ki-67 labeling for living cells with DAPI labeling of nuclei, followed by scanning for pimonidazole conversion fluorescence. Masson–Goldner trichrome labeling allows for identification of necrotic tissue (n) and surrounding living tumor. As shown in lower panels, conversion of pimonidazole in necrotic tissue was investigated for longer-term microtumors which had survived for 128 days after bioprinting and demonstrated necrotic formation surrounded by the living tumoral cells.

Next, it was essential to demonstrate that our long-time-established microtumors retain proliferative cells, as well as necrotic and hypoxic areas, as has long been known in colorectal tumors. The 3D bioprinted microtumors indeed display a necrotic center surrounded by proliferative cells, as shown with Masson–Goldner trichrome and KI-67 staining (Figure 6 upper panel). Hypoxic areas were confirmed with pimonidazole staining which is reductively activated in hypoxic cells and tissues (Figure 6). The specificity of this protocol which was carried out on tumors that had survived for 128 days after bioprinting is also used in vivo, so we believe that this gives additive information on tumor turnover and should allow for crossover comparisons to animal studies and perhaps eventually have the potential to replace them. Hypoxic areas are an important consideration in chemotherapeutic success since hypoxia mediates several mechanisms involved in treatment resistance and uncontrolled hypoxia following drug therapy is a significant reason for poor response to therapy [24,25].

### 3.4. Accurate Sophisticated Screening of Advanced Biologics Can Be Achieved in Microtumors

**Figure 7:**
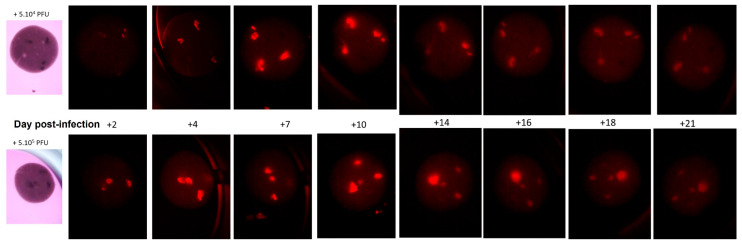
Virus-delivered chemotherapeutic response over time. Microtumors that had survived for 114 days after bioprinting and had been evaluated for continued tumor formation were subjected to oncolytic virus infection containing the FC1 enzyme-sponsored conversion of prodrug 5-FC to the active chemotherapeutic 5-FU. The virus successfully penetrated not only the printed model but also the microtumors. Maximum infection (mCherry labeling, in red) was noted after a few days, followed by a slow petering out as cells were killed by the 5-FU. The continual response after several days highlighted the bystander effect of the transfer of 5-FU throughout the tumor at different concentrations.

To further prove the validity of this model as a useful screening tool, we tested it with our advanced oncolytic virus (OV). The OV used in this study is derived from a vaccinia virus deleted for Thymidine Kinase/Ribonucleotide Reductase, and it expresses mCherry as an exogenous transgene model. mCherry allows the non-invasive monitoring of the infected cells with transgene expression by fluorescence microscopy. The mCherry expression was monitored over time in longer-living tumors that had survived for 114 days after bioprinting (Figure 7).

Targeting of the virus to the tumoral cells, noted over 21 days, shows the increased fluorescence of the labeled virus at the tumoral site, followed by a sharp reduction by Day 21, when the cells had been destroyed. For the slightly higher concentration of virus (lower panels, Figure 7), the fluorescence was slightly higher on Day 14, but by Day 21 there was little difference in the outcome in the cells, indicating that the two concentrations of the virus were not particularly limiting.

**Figure 8:**
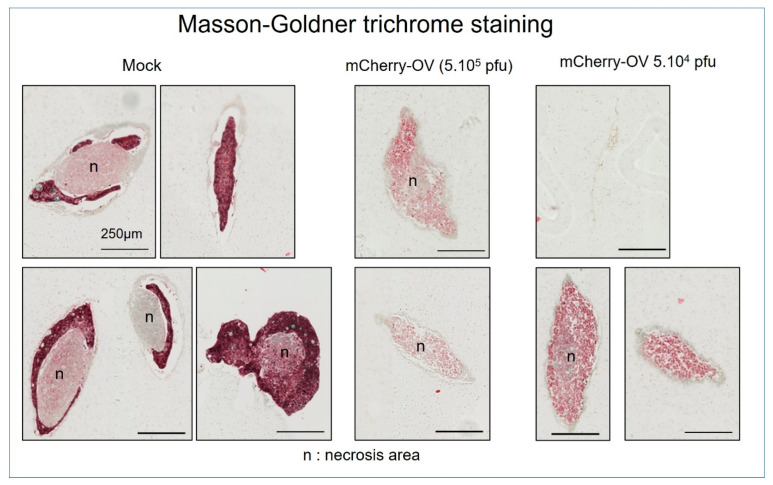
Histological analysis of OV impact on microtumors. The effective and direct delivery of chemotherapeutic agents by OV is demonstrated by the destruction of the surrounding living tumoral tissues and the exposure of the necrotic internal spaces. mCherry-OV delivery on right side panels and controls on the left.

Further analysis of the tumors by histology allowed for the visualization of the effects of the process of delivery of the oncolytic virus (Figure 8).

It was possible to view the outset of infection and expression in the first few days with an increasingly bright expression of mCherry and a slightly faster infection rate with the higher dose of 5 × 10^5^ PFU. Interestingly, all tumor clusters appear to be infected by Day 7 with both OV doses, indicating that even if the expression was dose-dependent on the first day, it would reach a similar level. With time and with both OV doses, the expression started to become more diffuse and to decrease, finally becoming very faint. Histologic analyses of the microtumors 25 days after infection reveal that the infected ones are totally necrotic and dead in comparison with the control condition which presents a necrotic area surrounded by a dense live cell area. Taken together, these results suggest that OVs are able to infect the microtumors, propagate and express their transgene for at least 21 days, at which point the expression shuts down due to the death of the tumor cells.

## 4. Discussion

It should be noted first that this study, carried out for around 6 months, was not on cell lines, but on primary human tissue and cells from an actual primary patient. The importance of this for developing longer-term research and/or screening strategies is of consequence since researchers have historically struggled with maintaining cell survival long-term. This gives some hope that such cultures will be available for long-term clonality studies and investigation of mutagenesis with time in these patients. The global failure rate of bringing new drugs to market is immense but exacerbated when it comes to advanced therapies. What can look good in simple standard experimentation often does not translate well to animal testing and on towards phased human clinical trials [26,27]. Problems in the treatment targeting the site of the cancer tumor rather than randomly around the human body are well known. Adding to this the multidrug resistance accumulation known in longer-term cancers makes for a difficult problem to overcome [28]. Even animal testing, although it has become more sophisticated with immunized gene-manipulated models and patient-derived xenograft models (PDX), has significant limitations in representing the human system. Failures often occur due to the size and the lifespan of animal models. A lack of a toxic reaction in the often-used murine system does not at all mean a successful treatment will be secured in the larger human organism. Genetics and immunology have a lot to do with this, but in cancer, metastatic and cellular dysregulation beyond the tumor is also important [29]. For these reasons, better humanized models are required in all stages of the development plan of new therapies to give better predictive results before clinical trials.

Already noted in the introduction are the advances towards organoid models. However, the variety of what an “organoid” is appears to be vast and requires some international classification to be agreed upon. We also employ organoids in our laboratories, but again, the main limitation is the nature of the short-lived testing platforms. Such quick testing is indeed important in the early stages of drug screening but not readily translatable for complex therapies and long-term outcomes in a patient [30]. For this, 3D complex models are increasingly required not only as cheaper tests to replace animals, but also to mimic the tumor microenvironment and growth in vivo as it changes dynamically. Potentially, as noted, this might allow for longer-term changes in the cancer cells with less animal testing, but it would require extensive and expensive studies.

Three-dimensional bioprinting, while still a science in development, can be a step forward over historical 3D models created with static scaffolds and microgravity bioreactors. Placement, more accurately, of cancer cells with or without additional cell types, adhesion molecules and cytokines can better represent the stages of tumor cell growth, from the early stages to large tumors self-encapsulating, hypoxic kinetics and metastatic events [31]. It is for this reason that we advanced our work in this direction. The creation of rapidly formed tumor models can allow for rapid screening of combinations of therapies that may have a suitable result in a particular patient before the patient risks treatment [32]. This level of personalized medicine may seem obvious but does not currently exist. The OncoDx test, which used historical patient and chemotherapy response data, was a huge step forward in treatments, not least in breast cancers, but is still not personalized. However, OncoDx has shown that better prediction is essential as part of the hospital process. We believe that what we have created for colorectal cancer does allow for something important to add to the treatment decision-making processes. Since our models were reproducible, used small parts of the tumor (with the remaining going to several diagnostic laboratories) and were creating in vitro models before the treatment schedule of the patient, there is a strong justification for considering therapy screening now before touching the patient. This would indeed be a seismic step forward.

Beyond what our models could do in the short term are the advantages of the longer-term growth we have seen (Figure 5, Figure 6, Figure 7 and Figure 8). Allowing the models to grow independently for 5 or 6 months or more can help to mimic what happens and provide research data on the mutation of the cancer cells over time, as well as helping in the investigation of multidrug resistance kinetics. In addition, longer-term models also allow us to investigate mechanisms of metastasis in patients and the reasons why the cells move away from the primary tumor, not least when the hypoxic levels in the center of the original tumor increase (Figure 6). Modeling of necrosis and apoptosis is important because they have a direct relation to the dysregulation of the local environment the tumor finds itself in and potentially the stimulation of new blood vessel development as the system works to overcome the detection of hypoxia and cellular damage. The circular tumor development seen in our models with hypoxic interiors was an important development in cancer modeling since organoid and short-term two-dimensional screening does not well evaluate that. With these models, in their more humanized form and more closely resembling tumors found in the patient, we can test the outcome of chemotherapy treatments at all stages, including late grades. Indeed, it is important to consider that 3D tumor environments may stimulate cellular changes, and although genetic screening and genomic work are ongoing for the cells of the current patient used in this study (IMODI Cancer Consortium), more studies are required.

Our OV testing was important for several reasons (Figure 7 and Figure 8). First, advanced therapies such as viral, antibody or Car-T-cell therapies are discussed often, but complex therapies still must penetrate through the system to the site of the tumor and then on into the tumor cells [33,34]. This is more challenging than it seems, particularly with tumors that form barriers, either fibrotic or cellular. Accumulation of immune cells at the site of tumors is often seen, and degradation of the local environment can additionally form a strong barrier to penetrate [35]. However, here we were able to verify the penetration of the virus into the tumor. The long-term expression of the OV suggests that in contrast to what had been observed in vitro in 2D cell culture, the virus does not kill the infected cells in a few days, but it will take more than 2 weeks, which probably mimics the clinical situation better. This longer time span of expression and killing will allow further studies to assess the expression of the therapeutic transgene by the OV, which could improve their efficacy. Another important area of drug failure in patients is the interstitial pressure between the tumoral cells themselves. In CRC as well as other cancers, including pancreatic cancer, the cells are so tightly bound through molecular interaction, channel formation and antigen control that the penetration of chemotherapies can be a significant limitation [36]. In our model, we were able to replicate this and show that OV-directed therapies would allow for penetration into this tight system and allow for tumor cell death directly and by the bystander effect (cell-to-cell transfer of chemotherapeutic molecules) [37,38]. Again, bioprinting allows this important advance in a way that organoids and other simpler systems cannot. The limitations in the model are the length of time required to grow the cells and the waiting time for testing, but since few systems other than animals can replicate this level of cellular organization, it is worth pursuing. Cells may mutate during this length of time, and this is work being investigated by us, but this could be also a useful tool to help researchers understand more about the longer-term changes that are stimulated in a 3D environment—particularly when apoptosis and necrosis are involved.

Longitudinal modeling of outcomes is one of the main reasons for the use of animal models. Therefore, our 3D bioprinted models provide an alternative of value to consider in colorectal cancer drug screening and a direct method of reducing animal testing with advanced humanized results.

## 5. Conclusions

Sophisticated humanized models are an important step in developing advanced chemotherapeutic and cellular-therapeutic strategies of the future. Three-dimensional bioprinted colorectal cancer tumors were successfully produced in this study and maintained for growth for between 1 day and more than 5 months. Short- and long-term growth was mimicked in vitro and provides an important cost-effective screening system not only for basic chemotherapies but also for advanced therapeutics, including the OV strategy we successfully employed here.

## Figures and Tables

**Table 1:** Characteristics of the patient investigated for this study. The patient chosen had primary colorectal adenocarcinoma with metastasis. After processing, the disaggregated purified tumor cells were processed immediately for use or amplified for further use. Typically, tumor sizes available for research are small, which is a limitation of cancer research.

Category	Result
Sex	Male
Age	74
Weight	80.78 kg
BMI	28.49
Diagnosis	Primary colorectal adenocarcinoma
Extent	Metastatic
Tumor sample size	3.66 g
Models printed per hour	173

## Data Availability

Data created in this study are available from the correspondence address provided.

## References

[1] Ingber D.E. (2022). Human organs-on-chips for disease modelling, drug development and personalized medicine. Nat. Rev. Genet..

[2] Russomano T., Cardoso R., Falcao F.P., Dalmarco G., dos Santos C.R.V., dos Santos L.G.F., de Azevedo D.F.G., dos Santos M.A., Martinelli L., Motta J.D. Development and Validation of a 3D Clinostat for the Study of Cells during Microgravity Simulation. Proceedings of the 2005 IEEE Engineering in Medicine and Biology 27th Annual Conference.

[3] Gupta S., Bit A. (2022). 3D bioprinting in tissue engineering and regenerative medicine. Cell Tissue Bank..

[4] Sung HFerlay F., Siegel R.L., Laversanne M., Soergomataram I., Jemal A., Bray F. (2021). Global Cancer Statistics 2020: GLOBOCAN Estimates of Incidence and Mortality Worldwide for 36 Cancers in 185 Countries. CA Cancer J. Clin..

[5] Siegel R.L., Miller K.D., Fuchs H.E., Jemal A. (2022). Cancer statistics, 2022. CA Cancer J. Clin..

[6] Loomans-Kropp H.A., Umar A. (2019). Increasing Incidence of Colorectal Cancer in Young Adults. J. Cancer Epidemiol..

[7] Creasy J.M., Sadot E., Koerkamp B.G., Chou J.F., Gonen M., Kemeny N.E., Balachandran V.P., Kingham T.P., DeMatteo R.P., Allen P.J. (2018). Actual 10-year survival after hepatic resection of colorectal liver metastases: What factors preclude cure?. Surgery.

[8] Engstrand J., Nilsson H., Strömberg C., Jonas E., Freedman J. (2018). Colorectal cancer liver metastases—A population-based study on incidence, management and survival. BMC Cancer.

[9] Crooke H., Kobayashi M., Mitchell B., Nwokeji E., Laurie M., Kamble S., McKenna M., Masood A., Korytowsky B. (2018). Estimating 1-and 5-year relative survival trends in colorectal cancer (CRC) in the United States: 2004 to 2014. J. Clin. Oncol..

[10] Chao C., Carmical J.R., Ives K.L., Ives K.L., Wood T.G., Aronson J.F., Gomez G.A., Djukom C.D., Hellmich M.R. (2012). CD133+ colon cancer cells are more interactive with the tumor microenvironment than CD133- cells. Lab. Investig..

[11] Reidy E., Leonard N.A., Treacy O., Ryan A.E. (2021). A 3D View of Colorectal Cancer Models in Predicting Therapeutic Responses and Resistance. Cancers.

[12] Shakibaei M., Kraehe P., Popper B., Shayan P., Goel A., Buhrmann C. (2015). Curcumin potentiates antitumor activity of 5-fluorouracil in a 3D alginate tumor microenvironment of colorectal cancer. BMC Cancer.

[13] Loessner D., Stok K.S., Lutolf M., Hutmacher D.W., Clements J.A., Rizzi S.C. (2010). Bioengineered 3D platform to explore cell-ECM interactions and drug resistance of epithelial ovarian cancer cells. Biomaterials.

[14] Maloney E., Clark C., Sivakumar H., Yoo K., Aleman J., Rajan S.A.P., Forsythe S., Mazzocchi A., Laxton A.W., Tatter S.B. (2020). Immersion Bioprinting of Tumor Organoids in Multi-Well Plates for Increasing Chemotherapy Screening Throughput. Micromachines.

[15] Jurga M., Dainiak M.B., Sarnowska A., Jablonska A., Tripathi A., Plieva F.M., Savina I.N., Strojek L., Lungvid H., Kumar A. (2011). The performance of laminin-containing cryogel scaffolds in neural tissue regeneration. Biomaterials.

[16] Mueller A.A., Forraz N., Gueven S., Atzeni G., Degoul O., Pagnon-Minot A., Hartmann D., Martin I., Scherberich A., McGuckin C. (2014). Osteoblastic differentiation of Wharton jelly biopsy specimens and their mesenchymal stromal cells after serum-free culture. Plast. Reconstr. Surg..

[17] McGuckin C.P., Jurga M., Miller A.M., Sarnowska A., Wiedner M., Boyle N.T., Lynch M.A., Jablonska A., Drela K., Lukomska B. (2013). Ischemic brain injury: A consortium analysis of key factors involved in mesenchymal stem cell-mediated inflammatory reduction. Arch. Biochem. Biophys..

[18] Sarnowska A., Jablonska A., Jurga M., Dainiak M., Strojek L., Drela K., Wright K., Tripathi A., Kumar A., Jungvid H. (2013). Encapsulation of mesenchymal stem cells by bioscaffolds protects cell survival and attenuates neuroinflammatory reaction in injured brain tissue after transplantation. Cell Transplant..

[19] Sbirkov Y., Molander D., Milet C., Bodurov I., Atanasov B., Penkov R., Belev N., Forraz N., McGuckin C., Sarafian V. (2021). A Colorectal Cancer 3D Bioprinting Workflow as a Platform for Disease Modeling and Chemotherapeutic Screening. Front. Bioeng. Biotechnol..

[20] Zhou XZhu W., Nowicki M., Miao S., Cui H., Holmes B., Glazer R.I., Zhang L.G. (2016). 3D Bioprinting a Cell-Laden Bone Matrix for Breast Cancer Metastasis Study. ACS Appl. Mater. Interfaces.

[21] Zhao Y., Yao R., Ouyang L., Ding H., Zhang T., Zhang K., Cheng S., Sun W. (2014). Three-dimensional printing of Hela cells for cervical tumor model in vitro. Biofabrication.

[22] Dai X., Ma C., Lan Q., Xu T. (2016). 3D bioprinted glioma stem cells for brain tumor model and applications of drug susceptibility. Biofabrication.

[23] Mencattini A., Lansche C., Veith I., Erbs P., Balloul J.-M., Quemeneur E., Descroix S., Mechta-Grigoriou F., Zalcman G., Zaupa C. (2022). Direct imaging and automatic analysis in tumor-on-chip reveal cooperative antitumoral activity of immune cells and oncolytic vaccinia virus. Biosens. Bioelectron..

[24] Jing X., Yang F., Shao C., Wei K., Xie M., Shen H., Shu Y. (2019). Role of hypoxia in cancer therapy by regulating the tumor microenvironment. Mol. Cancer.

[25] Aguilera K.Y., Brekken R.A. (2014). Hypoxia Studies with Pimonidazole in vivo. Bio-Protocol.

[26] Seyhan A.A. (2019). Lost in translation: The valley of death across preclinical and clinical divide—Identification of problems and overcoming obstacles. Transl. Med. Commun..

[27] Wong C.H., Siah K.W., Lo A.W. (2019). Estimation of clinical trial success rates and related parameters. Biostatistics.

[28] Van der Jeught K., Xu H.C., Li Y.J., Lu X.-B., Ji G. (2018). Drug resistance and new therapies in colorectal cancer. World J. Gastroenterol..

[29] Boutin A.T., Liao W.-T., Wang W., Hwang S.S., Karpinets T.V., Cheung H., Chu G.C., Jiang S., Hu J., Chang K. (2017). Oncogenic Kras drives invasion and maintains metastases in colorectal cancer. Genes Dev..

[30] Rios de la Rosa J.M., Wubetu J., Tirelli N., Tirella A. (2018). Colorectal tumor 3D in vitro models: Advantages of biofabrication for the recapitulation of early stages of tumour development. Biomed. Phys. Eng. Express.

[31] Augustine R., Kalva S.N., Ahmad R., Zahid A.A., Hasan S., Nayeem A., McClements L., Hasan A. (2021). 3D Bioprinted cancer models: Revolutionizing personalized cancer therapy. Transl. Oncol..

[32] Chen H., Cheng Y., Wang X., Wang J., Shi X., Tan W., Tan Z. (2020). 3D printed in vitro tumor tissue model of colorectal cancer. Theranostics.

[33] Cascinu S., Berardi R., Salvagni S., Beretta G.D., Catalano V., Pucci F., Sobrero A., Tagliaferri P., Labianca R., Scartozzi M. (2008). A combination of gefitinib and FOLFOX-4 as first-line treatment in advanced colorectal cancer patients. A GISCAD multicentre phase II study including a biological analysis of EGFR overexpression, amplification and NF-κB activation. Br. J. Cancer.

[34] Klemm F., Joyce J.A. (2015). Microenvironmental regulation of therapeutic response in cancer. Trends Cell Biol..

[35] Blondy S., David V., Verdier M., Mathonnet M., Perraud A., Christou N. (2020). 5-Fluorouracil resistance mechanisms in colorectal cancer: From classical pathways to promising processes. Cancer Sci..

[36] Wang T., Chen Z., Zhu Y., Pan Q., Lui Y., Qi X., Jin L., Jin J., Ma X., Hua D. (2015). Inhibition of transient receptor potential channel 5 reverses 5-Fluorouracil resistance in human colorectal cancer cells. J. Biol. Chem..

[37] Hoare O., Fraunhoffer N., Elkaoutari A., Gayet O., Bigonnet M., Roques J., Nicolle R., McGuckin C., Forraz N., Sohier E. (2021). Exploring the Complementarity of Pancreatic Ductal Adenocarcinoma Preclinical Models. Cancers.

[38] Inamura K. (2018). Colorectal Cancers: An Update on Their Molecular Pathology. Cancers.

